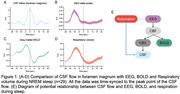# Respiratory volume changes and CSF circulation during human NREM sleep

**DOI:** 10.1002/alz.093200

**Published:** 2025-01-09

**Authors:** Jeyeon Lee, Seokbeen Lim, Mahathi Kandimalla, Sujala Ghatamaneni, Jay Thakkar, Sannidhi Dewan, Petrice M Cogswell, Jeffrey L. Gunter, Hugo Botha, Jonathan Graff‐Radford, Soonhyun Yook, Hosung Kim, Stuart J McCarter, John G Park, Erik K St. Louis, Carlos Mantilla, John Huston, Val J. Lowe, Hoon‐Ki Min

**Affiliations:** ^1^ Mayo Clinic, Rochester, MN USA; ^2^ Hanyang University, Seoul Korea, Republic of (South); ^3^ USC, Los Angeles, CA USA

## Abstract

**Background:**

Sleep’s crucial role in maintaining brain health is increasingly recognized, particularly due to the rising prevalence of neurodegenerative diseases. It not only supports cognitive function but also aids in clearing brain metabolic waste through cerebrospinal fluid (CSF) dynamics. During sleep, especially in the non‐rapid eye movement (NREM) phase, CSF flow increases, essential for removing neurotoxic substances like amyloid‐beta proteins. This study explores the link between sleep respiratory patterns and CSF flow promotion.

**Method:**

Electroencephalography (EEG), and functional magnetic resonance imaging (fMRI) were simultaneously acquired during sleep from Gu, et al. (n = 29) (Gu et al. 2022). Respiration, measured via a respiratory belt, calculated respiratory volume (RV) as the standard deviation within a 5 second sliding window. EEG data processed delta band power (1‐4Hz) averaged across channels. Parcellated fMRI data using the brain template followed by the calculation of the averaged gray matter (GM) blood‐oxygen‐level‐dependent (BOLD) signal using the GM mask. CSF inflow was manually extracted from the fMRI’s bottom slice at the foramen magnum level. Subsequently, all signals were temporally aligned to the peak of the CSF inflow signal.

**Result:**

At the time of CSF inflow (time = 0 sec. in the Figure 1A‐D), we observed an earlier increase in RV and EEG delta power, followed by changes in the GM BOLD signal (Figure 1). EEG and RV changes preceded the BOLD signal changes, which, in turn, preceded changes in CSF flow. Sensitivity to detect RV involvement during MRI varied with the placement of the respiratory belt, being least sensitive on the chest and more on the abdomen.

**Conclusion:**

These findings raise an interesting question on the role respiratory patterns during sleep, particularly in the NREM phase. Speculatively, RV change may influence CSF circulation during NREM sleep (Figure 1E). This enhancement in CSF flow is crucial for the clearance of metabolic waste from the brain, potentially reducing the risk of neurodegenerative diseases. Our findings indicate that respiratory volume may serve as a significant marker for CSF circulation during sleep.